# Soil organic matter is essential for colony growth in subterranean termites

**DOI:** 10.1038/s41598-021-00674-z

**Published:** 2021-10-28

**Authors:** Aaron Mullins, Thomas Chouvenc, Nan-Yao Su

**Affiliations:** Department of Entomology and Nematology, Ft Lauderdale Research and Education Center, Institute of Food and Agricultural Sciences, University of Florida, 3205 College Ave, Ft Lauderdale, FL 33314 USA

**Keywords:** Biochemistry, Chemical biology, Ecology, Evolution, Microbiology, Physiology

## Abstract

Intrinsic dinitrogen (N_2_) fixation by diazotrophic bacteria in termite hindguts has been considered an important pathway for nitrogen acquisition in termites. However, studies that supported this claim focused on measuring instant N_2_ fixation rates and failed to address their relationship with termite colony growth and reproduction over time. We here argue that not all wood-feeding termites rely on symbiotic diazotrophic bacteria for colony growth. The present study looks at dietary nitrogen acquisition in a subterranean termite (Rhinotermitidae, *Coptotermes*). Young termite colonies reared with wood and nitrogen-rich organic soil developed faster, compared to those reared on wood and inorganic sand. More critically, further colony development was arrested if access to organic soil was removed. In addition, no difference of relative nitrogenase expression rates was found when comparing the hindguts of termites reared between the two conditions. We therefore propose that subterranean termite (Rhinotermitidae) colony growth is no longer restricted to metabolically expensive intrinsic N_2_ fixation, as the relationship between diazotrophic bacteria and subterranean termites may primarily be trophic rather than symbiotic. Such reliance of Rhinotermitidae on soil microbial decomposition activity for optimal colony growth may also have had a critical mechanistic role in the initial emergence of Termitidae.

## Introduction

Phytophagous insects have adapted various strategies to acquire and retain nitrogen for growth and reproduction^1^. For example, aphids feed extensively on plant phloem with low nitrogen content and produce honeydew as a way to excrete excess carbon, while sequestering nitrogen^2^. Termites are also a nitrogen-limited group, and as a result have evolved a number of distinct characteristics regarding nitrogen conservation and acquisition. These include eusociality, which allows for nitrogen recycling at the colony level^3,4^, and relatively small bodies compared to their wood roach ancestors^5^. Termite feeding guilds that primarily consume sound wood have access to an ecological niche with little competition, owing to their peculiar ability to unlock energy stored as cellulose polymers^6^. All “lower” termites, which are phylogenetically basal^7,8^ rely on a host of symbiotic Protozoa for cellulose digestion^9^. However, wood is intrinsically poor in nitrogen (up to 0.15%)^4,10,11^, and such termite colonies rely on alternative sources to acquire sufficient nitrogen for optimal colony growth and reproduction. Comparatively, in Termitidae (i.e. “higher” termites), which have lost their mutualistic protozoa and primary rely on bacteria for cellulose digestion, many species have switched to a “soil” diet with access to nitrogen-rich organic residues, leading to relatively high concentration of ammonia in their guts^8,12–14^. Therefore, throughout the evolution and radiation of Neoisoptera^15^, the optimal use of soil resources was possibly one of the key behavioral changes that allowed for the higher diversity and ecological dominance of the Termitidae^14,16^.

While access to relatively nitrogen-rich soil organic matter (soil OM) for colony growth is no longer a limiting factor in many Termitidae, “lower” termites still primarily or exclusively rely on a wood diet, and therefore evolved within a nitrogen-limited context^17^. One adaptation employed by lower termites to acquire usable nitrogen is to rely on symbiotic nitrogenase-producing diazotrophic bacteria in their hindgut through fixation of atmospheric N_2_^18,19^. In 1973, both Breznak et al.^18^ and Benemann^20^ independently reported intrinsic N_2_ fixation in the hindgut of termites, which was subsequently confirmed in various termite species^21–24^. However, results confirming the importance of N_2_ fixation in the termite gut have been problematic^9^ as estimates ranged from 0 to 60% of all nitrogen in the termite’s body being of “recent” atmospheric origin, and the two methods for measuring or confirming N_2_ fixation rates, the acetylene reduction assay^25^ and ^15^N_2_ stable isotope assays^26^ present inherent methodological limitations^27–29^.

It has been proposed that a problem fundamental to most insect/diazotroph investigations is that they primarily focus on only one of three aspects of the phenomenon^30^. These aspects are: (1) determining the presence of diazotrophs; (2) measuring nitrogen fixation rates; and (3) measuring the assimilation of newly fixed N into insect tissues. There are very few investigations across the insects that address all three aspects^30^. While N_2_ fixation studies are relatively abundant for the termites, they suffer from the same lack of all three aspects. In addition, there are a few major problems specific to N_2_ fixation studies in termites with complex social systems. First, they primarily employed the use of a field-collected foraging population under highly contrived laboratory conditions. Such studies only include one cohort of a termite colony, ignoring the dynamics of nitrogen flow within the whole colony among different castes, life stages, and task allocations^4,31^. Second, all of these studies measured N_2_ fixation rates in very short intervals (hours or days), which could not be correlated with colony growth over time. Third, ^15^N_2_ isotope studies run the risk of a number of potential errors: fractionation due to tissue-level degradation^27^, contamination of enriched ^15^N_2_ sources^28^, pressure-related nitrogen solubilization in tissues, or even a reflection of prior trophic-level feeding^29,32^. Finally, atmospheric N_2_ fixation has a high energetic cost: roughly 20 ATP are hydrolyzed to release 1 N from N_2_^33^.

This last point reveals a potential discrepancy on the biological relevancy of the use of energetically expensive diazotrophic bacteria gut mutualists across lower termites. Some termites with slow metabolism and small colonies such as drywood termites (Kalotermitidae) may be able to offset the energetically expensive application of symbiotic nitrogenase from the abundance of sugar resulting from cellulose digestion^33^. However, some of the most phylogenetically derived lower termites within Rhinotermitidae have the ability to grow colonies up to millions of individuals within a few years and reach a critical biomass prior to investing in the production of alates^34,35^. We here question how relevant such an inherently inefficient nitrogen acquisition pathway would remain their primary source of supplementary nitrogen for such large colonies. The *Coptotermes-Reticulitermes-Heterotermes* clade (within the paraphyletic Rhinotermitidae) represents a transitional group within the evolution of termites, as it is the sister group to all Termitidae, while having retained their cellulositic protozoa mutualists^8,14^. Contrary to Kalotermitidae, most species within these subterranean termite genera have a highly efficient foraging strategy through soils (> 100 m foraging distance)^36,37^ and many of them have a pest status^38–40^. It was suggested that the soil foraging behavior was present in the common ancestor to Termitidae and *Coptotermes-Reticulitermes-Heterotermes* and ultimately allowed Termitidae to acquire alternative soil microbes (bacteria or fungi) for the digestion of cellulose^14^. However, we here argue that this access to the soil also provided an alternative source of usable nitrogen, which was possibly instrumental in the improved termite colony growth in Rhinotermitidae.

Because previous studies primarily focused on instant N_2_ fixation rates, and not on how such N_2_ fixation translates into colony growth, our current understanding of the nitrogen flow in wood-feeding termite colonies is based on potentially erroneous extrapolations. Since the earlier part of the twentieth century, there has been no study attempting to correlate atmospheric N_2_ fixation rates with growth and reproduction in termite colonies. However, within the half-century between Cleveland^41^ initially predicting N_2_ fixation in termites and its actual discovery in 1973^18,20^, three studies published between 1932 and 1941 did a total nitrogen inventory taken in *Zootermopsis* before and after termite colony growth in laboratory conditions^42–44^. These studies remarkably did not find a net increase in total nitrogen after rearing colonies on wood or filter paper alone, and interpreted their results as atmospheric N_2_ fixation not being a significant source of nitrogen for colony growth. A more recent nitrogen inventory study with young colonies of *Coptotermes formosanus* (Shiraki) also supported the view that colony growth cannot be sustained by solely relying on fixation through mutualistic diazotrophic gut bacteria^45^. Breznak et al.^18^ also reported that when the cellulose diet was enriched with NH_4_^+^ or NO_3_^−^, the measured N_2_ fixation rates in termite guts reduced in direct relation to the amount of nitrogen added to their diet^18^. Finally, it was suggested that some termite species may acquire nitrogen both through symbiotic N_2_ fixation and selective foraging within their behavioral repertoire^46^. Such combined observations lend credence to our hypothesis that while some termite species may be able to fix atmospheric N_2_ in their hindgut as means of primary nitrogen procurement, other termites may forego this metabolically expensive process when alternative, dietary sources of nitrogen are available for significant colony growth.

After a decade of rearing laboratory *C. formosanus* colonies from a pair of alates to maturity, we have noticed improved colony growth when given access to nitrogen-rich soil OM. In addition, dark soil was often observed in the gut of both laboratory and field collected *C. formosanus*, suggesting that termites are able to ingest soil particles. More recently, a study comparing metagenomic profiles between termites exhibiting one-piece and extended life types suggested that dietary nitrogen may be utilized as a supplement to an all-wood diet^47^. These observations beg the question of whether nitrogen-rich soil OM is a primary source of nitrogen for a growing colony of *C. formosanus*. The objective of the present study is to measure colony growth and total nitrogen present in colonies of subterranean termites (*C. formosanus)* in either the presence or absence of nitrogen-rich soil OM over a period of 20 months after foundation, in order to determine if subterranean termite colonies can optimally grow in the absence of dietary nitrogen. We also tested if colonies are able to maintain their growth if their access to soil OM was temporarily removed.

During initial experimental design, our intent was to include soils with different nitrogen levels as experimental groups. However, this idea was abandoned as we couldn’t find a reliable method for amending soil nitrogen content without drastically affecting not only the survivability of the termites, but also standardizing the micronutrient composition of the soil provided. The chosen alternative was to instead include a molecular assay as a way of measuring termite physiological response to different nitrogen levels between only two groups, those with access to soil and those without. The relative nitrogenase expression of the termite hindgut fauna was measured among treatments in order to determine if the nitrogen acquisition is directly acquired through the diet or indirectly acquired through ingested micronutrients present in the soil. It could be argued that increased access to nitrogenase cofactor metals may affect productivity of diazotrophic gut bacterial mutualists. Thus any increase in nitrogen following colony growth could be the result of increased nitrogen fixation in worker hindguts, rather than of dietary origin. A nitrogenase expression assay between the two groups would show whether or not access to soil-borne cofactors affected overall nitrogenase activity.

## Results

### Incipient colony growth (0–14 months)

After 14 months of initial development, incipient termite colonies reared in sand had similar rates of success, (46%) than termite colonies reared in organic soil (63%) (χ^2^ = 2.68, *P* = 0.10) (success is here defined as a colony reared from two reproductive individuals surviving colony foundation with all castes present). However, colonies reared in organic soil had a biomass 2.65 times larger than colonies reared in sand. All colony growth metrics were larger for colonies reared in organic soil than colonies reared in sand for 14 months (Table 1). In addition, the weight of kings was similar in both treatments (6.50 ± 0.49 mg in organic soils, 6.61 ± 0.72 mg in sand, *t* test, *P* = 0.58). However, queen weight was significantly higher in organic soils (8.36 ± 0.44 mg) than in sand (7.9 ± 0.41 mg, *t* test, *P* = 0.001).

**Table 1:** Incipient termite colony growth (0–14 months). For each colony growth variable (mean ± SD), the same letter indicates no significant difference (χ^2^, or *t* test, α = 0.05).

Soil treatment	Successful colonies	No of eggs	No of larvae	No of workers	No of soldiers	Total colony biomass (mg)
Sand	22/48a	0.50 ± 1.10a	3.00 ± 3.15a	31.89 ± 3.58a	6.22 ± 1.31a	69.60 ± 5.61a
Organic soil	30/48a	5.58 ± 7.10b	9.33 ± 6.92b	85.87 ± 22.43b	11.92 ± 4.06b	184.30 ± 42.45b

### Colony growth between 14 and 20 months

**Figure 1:**
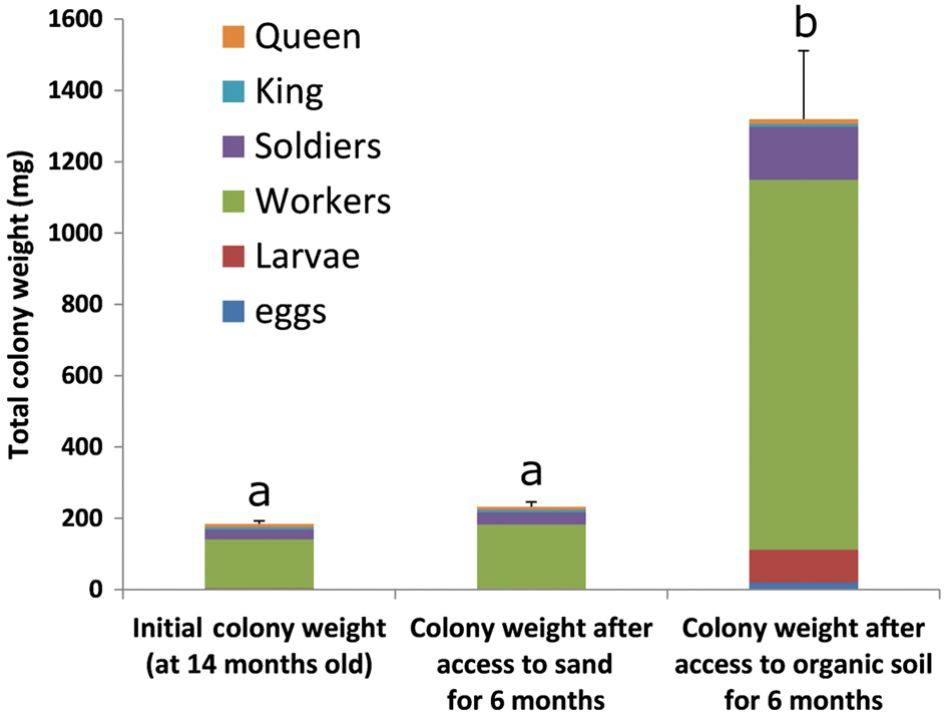
Average total colony weight (mean ± SD), after initial colony census (14 month-old colonies), after exposure to either sand, or organic soil for a period of 6 months (20 month-old colonies). Different letters indicate a significant difference in colony weight (Anova, Tukey post hoc, df = 2, F = 27.6, *P* < 0.001).

**Figure 2:**
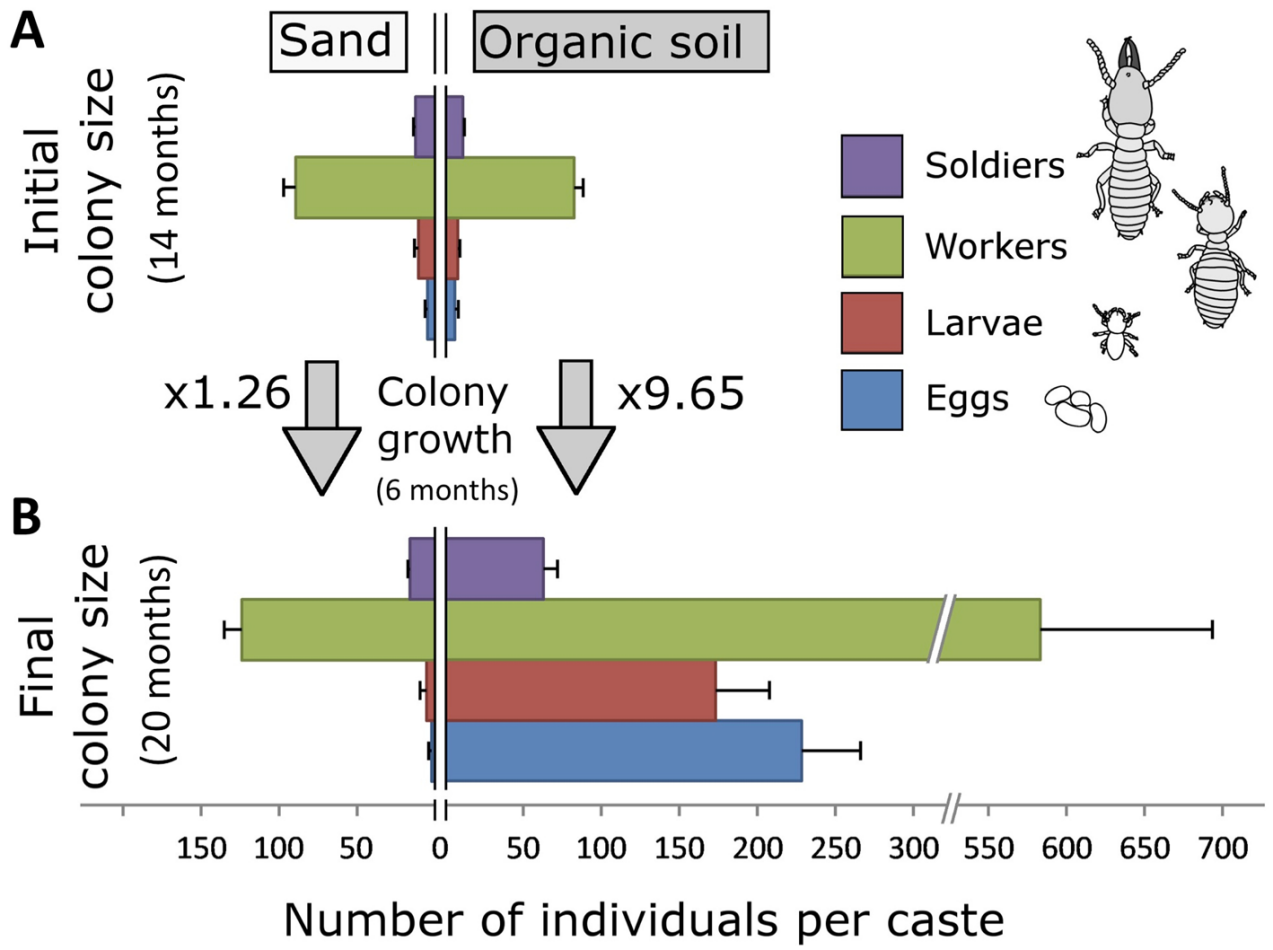
Colony size at (**A**) 14 months and (**B**) 20 months after two soil treatments. (average number of individuals ± SE, king and queen not presented). Total colony growth in sand condition was marginal (× 1.26 population increase, *P* = 0.032, *t* test), while substantial in organic soil (× 9.65 population increase, *P* < 0.001, *t* test). Comparisons of the average number of individuals for each independent castes at 20 month were all significantly higher in organic soil than in sand (*t* test, α = 0.05).

**Figure 3:**
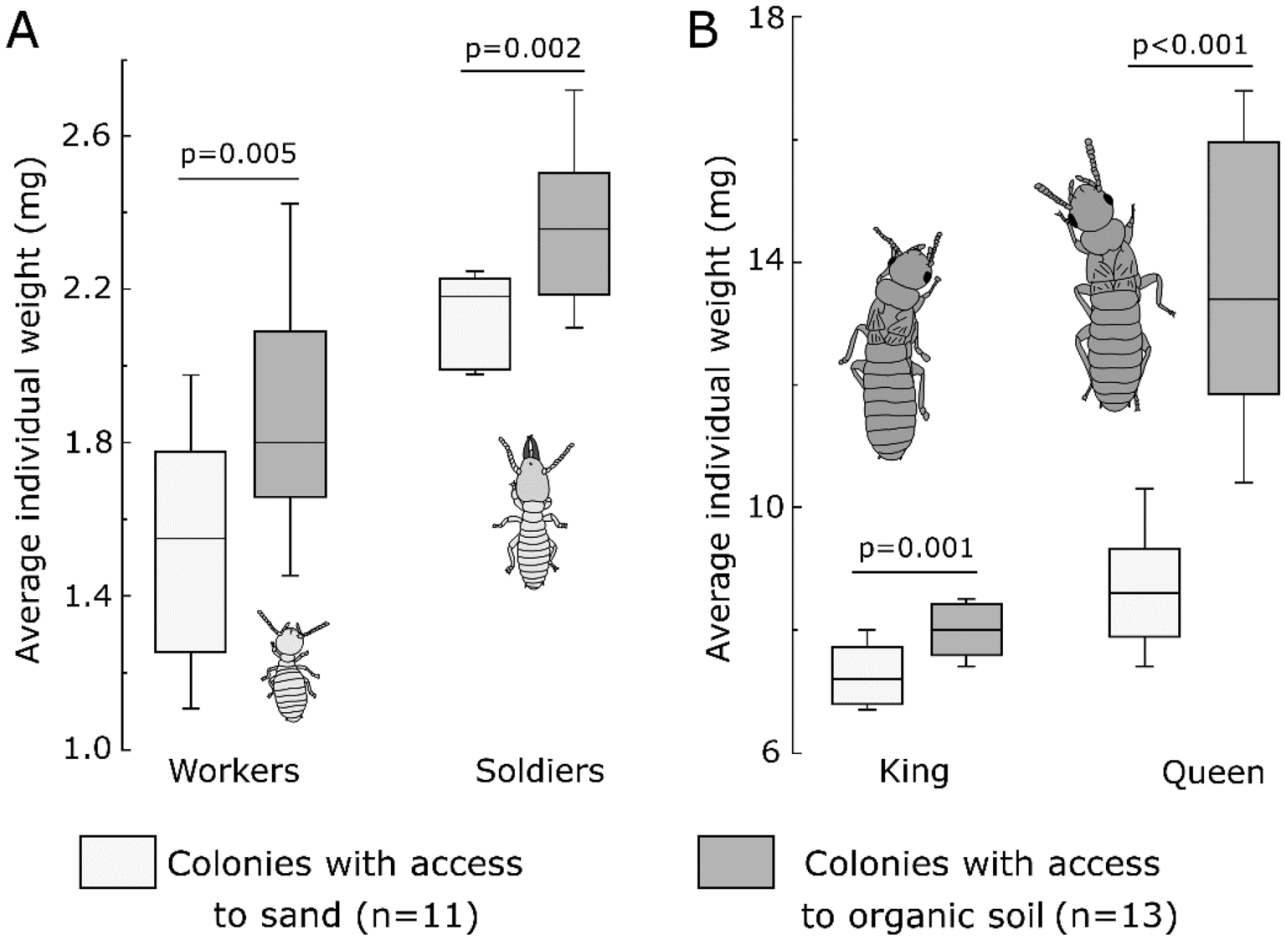
Boxplot distribution of individual caste weights (wet weight, mg) for 20 month-old colonies, after 6 months of soil treatment (sand vs. organic soil). (**A**) Workers and soldiers. (**B**) King and queen. All castes were significantly heavier in colonies reared on organic soil compared to colonies reared in sand (*t* test).

**Figure 4:**
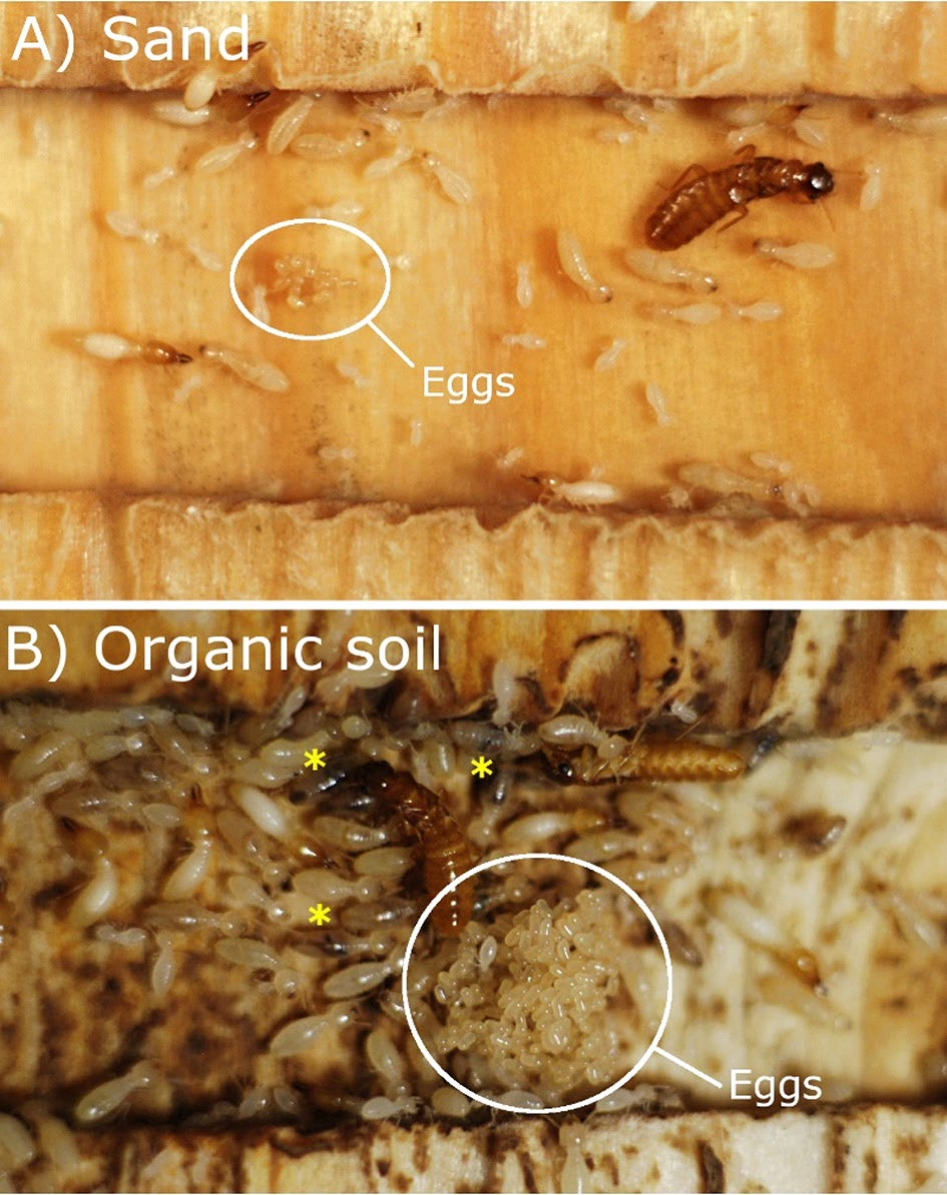
Photographs of the most successful *C. formosanus* 20 month-old colonies (partial view including the egg mass) when kept in a container with access to wood and (**A**) sand, or (**B**) organic soil, for a period of 6 months. *Examples of termites with dark organic soil visible in the gut.

**Figure 5:**
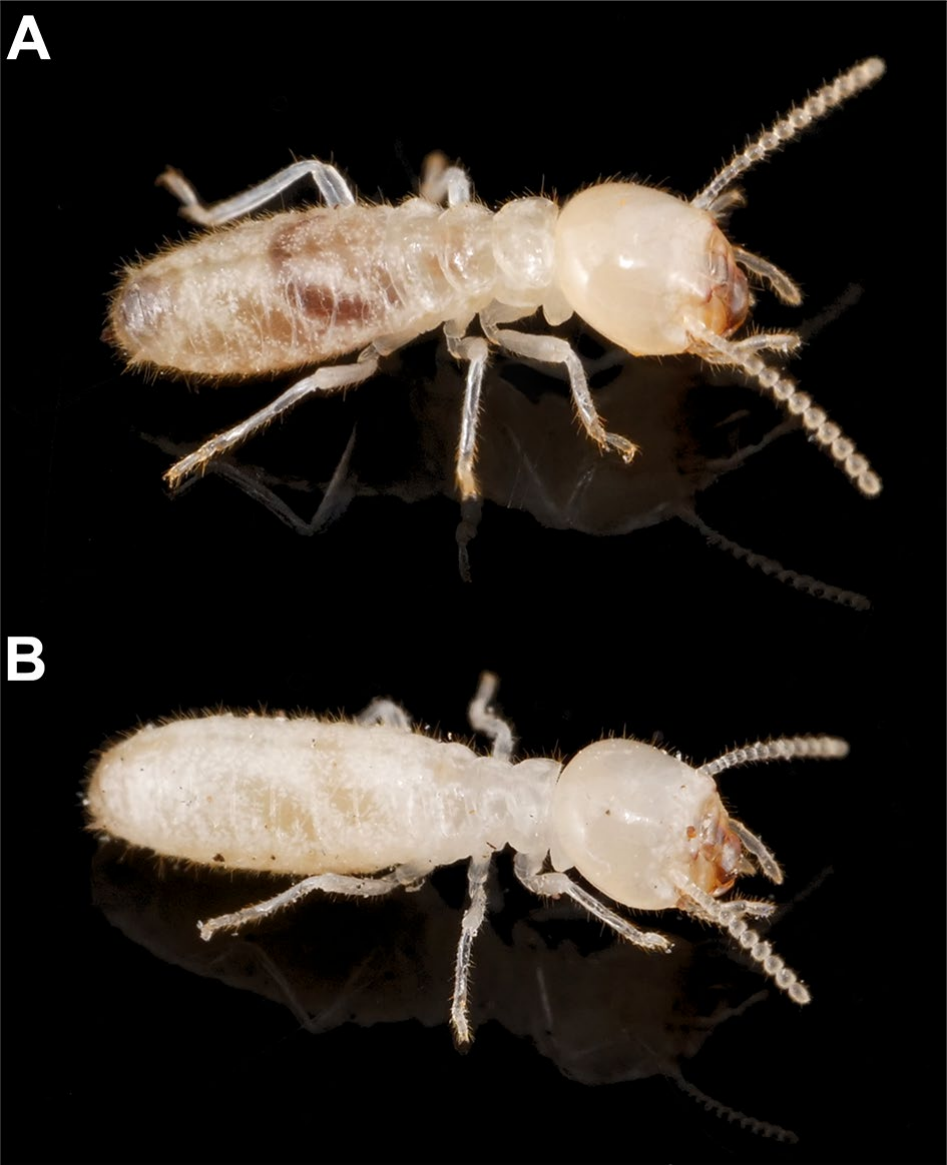
Photographs showing individual termite gut content as visible through the cuticle in termites reared with access to organic soil (**A**). The dark soil organic matter is clearly visible in the gut of the termite as opposed to those reared on only sand and wood (**B**).

The 30 successful colonies reared in organic soil for 14 months were then used for a subsequent bioassay. Half of them had maintained access to such organic soil and wood, while the other half only had access to sand and wood, for an additional 6 months of colony development. At 20 months, colonies from both soil treatments had similar survival rates (11/15 for sand, 13/15 for organic soil, χ^2^ = 0.83, *P* = 0.36). However, colonies that had maintained access to organic soil grew significantly more than those reared in sand during the 6-month period, as the biomass of colonies kept in sand did not significantly change over time (Fig. 1). Colonies reared in organic soils had a higher number of individuals (in all independent castes) than colonies reared in sand by a factor of 9.65 and 1.26 respectively (Fig. 2). All individual castes were heavier in colonies reared on organic soil (Fig. 3). Notably, queens from colonies reared in organic soil initiated their physogastric development (13.72 ± 2.12 mg) while queens from colonies reared in sand maintained a weight (8.69 ± 0.91 mg) equivalent to their pre-treatment weights at 14 months (8.36 ± 0.44 mg, *t* test, *P* = 0.08). Finally, workers in colonies reared on organic soil displayed visible dark soil particles in their gut, while workers from colonies reared on sand did not (Figs. 4, 5).

### Nitrogen content

We first confirmed the nitrogen content of each substrate used in this study: wood = 0.05 ± 0.005%, sand = 0%, and organic soil = 1.5 ± 0.02% (dry weight). For each soil type, the elemental analysis of termites from three 14 month-old and 20 month-old colonies, revealed that the nitrogen content represent ~ 2% of the wet biomass (~ 8% dry biomass) of whole colonies, regardless of rearing conditions (*t* test, *P* < 0.35). This translated into larger nitrogen quantities acquired by colonies reared on organic soils compared to colonies reared on sand, owing to their larger colony size (Table 2). The colony biomass did not change significantly in colonies reared in sand between 14 and 20 months, and as nitrogen content is constant at ~ 2% of the wet biomass, we confirmed that such colonies did not gain any additional nitrogen during this 6-month rearing period.

**Table 2:** Total termite colony nitrogen (mg) present in all castes of 20 month-old *C. formosanus* colonies after being reared for 6 months on either sand or organic soil (mean ± SD).

Soil treatment	Successful colonies	Eggs	Larvae	Workers	Soldiers	Colony total
Sand	11/15	0 (no eggs)	0.01 ± 0.02a	3.46 ± 0.4a	0.84 ± 0.11a	4.72 ± 0.50a
Organic soil	13/15	0.94 ± 0.68	1.79 ± 1.74b	17.89 ± 4.49b	3.17 ± 1.09b	24.33 ± 6.49b

For each variable, the same letter indicates no significant difference in total colony nitrogen content (*t* test, α = 0.05).

### Hindgut nitrogenase expression

Relative expression rates, (2^−Ct^ values) of nitrogenase (*nifH*) in worker hindguts did not differ significantly between termites reared on soil (1.12 ± 0.56) or sand (1.09 ± 0.46) (Student’s *t* test at = 0.05, *P* = 0.9538.), implying that N^2^ fixation rates in termite guts are identical between the two diet types.

## Discussion

Termite colonies grow by increasingly producing new individuals, which implies that in young colonies, there is a constant need for increasingly more resources to develop eggs and larvae into functional workers or soldiers^35^. In wood-feeding termites, carbon is not a limiting factor for such growth; however, our study demonstrated that a lack of access to dietary nitrogen is a fundamental limiting factor for initial colony growth in *C. formosanus*. Incipient subterranean termite colonies developed slowly when denied access to organic soil. In comparison, access to nitrogen-rich organic soil allowed termite colonies to reach a relatively large size within 14 months after foundation (~ 5.15 times the biomass). However, if these successful termite colonies were subsequently deprived access to organic soil at 14 months, colony size and total nitrogen content remained stagnant over the following 6 months. Colonies that had sustained access to organic soil during this time were able to initiate logarithmic growth^48^, and reached more than a thousand individuals by 20 months, with queens initiating physogastry (increase of ovary size for augmented reproductive capability)^35^. It has been suggested that subterranean termites rely on soil feeding for the acquisition of micronutrients^49^. Nitrogenase enzymes often utilize micronutrient metals such as molybdenum and vanadium^50–52^. Because of this, we suspected that lack of access to soil micronutrients may be a limiting factor to intrinsic nitrogenase activity of termite diazotrophic gut bacterial mutualists, and any increase in nitrogen following colony growth could be the result of increased nitrogen fixation in worker hindguts, rather than of dietary origin. This may well be true with one-piece nesting termites without access to soil such as most Kalotermitidae. However, the current study suggests that the rate of nitrogen fixation in *C. formosanus* is independent of their access to soil micronutrients. Instead, the relatively high nitrogen content of soil OM provides an easy and assimilable dietary source of nitrogen, and primarily explains the drastic growth of colonies that were able to feed on soil OM. Therefore, some of the free-living diazotrophs in the soil may simply be present in termite’s gut because of the direct consumption of soil rich in diazotroph diversity. Such observations of edaphophagy may be casual to “lower” termite species that have a life-type with recurrent contact to soils, as in addition to *Coptotermes*, we previously observed soil particles present in the hindgut of *Zootermopsis* (Archotermopsidae), *Neotermes* (Kalotermitidae), *Reticulitermes*, *Heterotermes*, and *Prorhinotermes* (Rhinotermitidae).

The presence of diverse diazotrophs and N_2_ fixation processes in the gut of termites is irrefutable^53–55^; however, our results suggest that their role as a primary source of nitrogen for colony growth may vary greatly among different termite feeding guilds. For example, most termite species in the family Kalotermitidae, which is phylogenetically basal to Rhinotermitidae^7,8^, live in a single piece of dry wood and have an extremely limited diversity of resources with no access to soils^6^. For these termites, dietary nitrogen intake comes exclusively from the wood they live in and feed on (poor nitrogen source) and colony growth may primarily rely on intrinsic atmospheric nitrogen fixation in their hindgut^9^. The cost of metabolically expensive N_2_ fixation as their primary means of nitrogen procurement is reflected by their relatively slow colony growth, limited colony size, and reduced reproductive output. In comparison, *C. formosanus* (Rhinotermitidae), with unrestricted access to water, micronutrients and nitrogen-rich soils^6^, may grow to millions of individuals as mature colonies and can produce massive dispersal flights^56^). The colony growth data obtained over a 20-month period unambiguously show that *C. formosanus* cannot solely rely on intrinsic N_2_ fixation for optimal colony growth and reproduction.

Although partial intrinsic N_2_ fixation still occurs through diazotrophic symbionts in *C. formosanus*^18^, it appears insufficient to account for colony growth, and did not vary depending on the termite diet. However, colonies reared on inorganic sand between 14 and 20 months did not lose biomass or nitrogen content, as they simply maintained the same population with marginal growth. It is important to note that these colonies did not experience a significant loss of individuals or biomass, implying an equivalent natality-mortality rate within this time frame, resulting in a stagnant population size. The fact that there is unavoidable loss associated with nitrogen economy of any insect system, through excretion, cannibalism, volatilization, or denitrification of nitrogenous compounds^57^, suggests that intrinsic N_2_ fixation in *C. formosanus* may still be relevant for temporary colony maintenance and survival in case of sudden or temporary loss of access to dietary nitrogen beyond wood. Such a system may therefore prevent rapid colony collapse in temporally-limited nitrogen resource conditions and offers time for the colony to forage for high-quality resource locations and re-initiate colony growth and reproduction. Arrested colony growth has often been attributed to spatial restrictions of laboratory-kept colonies^58–61^; however, our results suggest that lack of resources in the form of nitrogen-rich organic matter may also be one of the causes of arrested colony developmental growth^45,61^. This ability to maintain a colony size despite limited or no access to dietary nitrogen may also provide the ability of an invasive species such as *C. formosanus* to temporarily survive in boats and spread through maritime activity^62–66^.

Regarding the initial source of nitrogen in termites, it was suggested that essential amino acids present in subterranean termite tissues were primarily of bacterial origin^67^. In light of our result, there is a possibility that the majority of the N_2_ fixation by bacteria occurred in the soil the termites consumed (extrinsic), rather than in the termite gut (intrinsic). The present findings suggest that the relationship between young *Coptotermes* colonies and diazotrophic bacteria may be more of a transient interaction through a trophic relationship rather than a symbiotic one: subterranean termites directly feed on microorganisms and their metabolic output, by simply ingesting soil OM. In fact, Hungate^44^ provided unique elements that support our view. Hungate^44^ investigated total nitrogen available in wood, soil and termite biomass, before and after colony growth of *Zootermopsis nevadensis* Hagen. He reported a depletion of nitrogen in the soil, and suggested that the nitrogen from the soil was transported into the wood via wood-decomposing fungi and thus supplementing the diet of the termites, as termites would feed on such decomposed wood. However, he was puzzled at the fact that the mass of the soil itself had reduced significantly. Surprisingly, Hungate^44^ never mentioned the possibility that the termites may have been consuming nitrogen from the soil by directly ingesting soil OM. In initial experimental design for this study, attempts were made to measure the total mass of soil before and after the growth period, however it proved impossible due to the inability to separate soil from feces and transfer of substrate throughout the experimental chamber. However, given Hungate's extended growth times, we believe the same reduction in soil mass would have been observable given more growth time. While many termite species may preferentially feed on decomposing wood in order to increase their nitrogen intake^43^, our present study suggests that termites that have direct access to soils are able to bypass intermediate microbial decomposition processes involved in nitrogen flow, and directly ingest nitrogen-rich soil layers, as it represents the final byproduct of plant decomposition from various soil microorganisms, including soil diazotrophic bacteria and fungi.

One may argue that the difference in termite colony growth may partially be attributed to the soil providing other nutritional requirements, such as micronutrient or rare elements, as independent growth factors or cofactors of the N_2_ fixation metabolic pathway inside the termite gut^52^. Such argument cannot stand in light of *C. formosanus* colonies inability to grow in absence of substantial dietary nitrogen intake, which implies that their intrinsic N_2_ fixation ability is not as efficient as in Kalotermitidae, which grow from feeding solely on wood. However, the question remains as to why subterranean termites would fully maintain such an expensive symbiotic association when there is a reliably present and accessible nitrogen resource in the form of soil OM^68^? Although mutualistic associations provide reciprocal benefits to partnering organisms, there are often evolutionary costs and risks associated with such benefits^69^. Upon changing evolutionary pressures, the lack of flexibility of a symbiotic system may result in extinction, or breakdown of symbiosis^70^. The termite/protist/diazotroph model is no exception. Termitidae, the sister group of the *Coptotermes* clade, abandoned such a symbiotic system with a majority of species having switched to a soil-feeding habit^8,14^. In lower termites, protists and their own prokaryote symbionts are “locked into” obligate mutualism within the termite’s gut and cannot go back to a free-living form^70^. However, while subterranean termites are unable to live without the cellulolytic contribution of hindgut protists, they are able to obtain dietary nitrogen from soil, essentially bypassing their dependence on gut diazotrophic bacterial symbionts as a source of nitrogen. Subterranean termites may have lost the need to maintain the integrity of such expensive symbiosis, potentially making these diazotroph symbionts vestigial hold-overs.

Our observation may have deep implications on how Termitidae evolved in the first place. In a recent review, Chouvenc et al.^14^ discussed two potential scenarios for the loss of protozoa in Termitidae, one being the externalization of the digestion, farming soil microbes as a source for cellulose degradation, and the second one is the switch to a soil feeding habit where bacteria recruited from the soil became termite gut residents and replaced the function of protozoa. In both cases, the loss of protozoa implies an access to soil microbes for cellulose digestion. However, our study also suggests that the ecological dominance of Termitidae may also be the result of an unrestricted access to dietary nitrogen, where the ancestor to Termitidae lost its dependence from protozoa, by acquiring alternative mutualists from their soil environment.

In conclusion, while Cleveland^41^ in 1925 first predicted the possibility of intrinsic N_2_ fixation in the termites, subsequent studies in the early and mid-twentieth century that were looking at colony growth failed to demonstrate it, and most concluded that it did not take place^42–44^. Technological advances allowed for direct measurement, and the phenomenon was proven in termites in 1973^18,20^. However, the importance of such N_2_ fixation on colony development and reproduction has since remained inconclusive^9^. Paradoxically, no study considered the possibility of alternative nitrogen procurement in the form of nitrogen-rich soil OM, as most studies focused on fixation processes, and not on colony growth^9^. While most wood-feeding termites with one-piece nesting type (i.e. Kalotermitidae) can depend heavily on N_2_ fixation in their gut as a significant source of nitrogen for colony growth, for lack of alternative sources, our study shows that termite species with access to soil primarily rely on alternative, dietary sources of nitrogen that are readily available in their soil environment, which may have relaxed selective pressures on diet requirements and opened the door for the subsequent emergence and diversification of Termitidae^14^.

## Methods

### Colony growth assays

Incipient colonies of *C. formosanus* were initiated in June 2014 by pairing field-collected alates in rearing vials (37 cm^3^)^35^. Ninety-six rearing units were created, with 48 units containing white inorganic sand (8 cm^3^), wood (*Picea sp.* pieces, 10 cm^3^), 3% agar (15 cm^3^, for sustained moisture supply), and 48 units containing nitrogen-rich soil OM, wood and agar (in identical proportions). Three subsamples were taken each from the sand, wood and soil OM stock supplies used in this study and N content was measured in a CHN analyzer (Perkin Elmer, Waltham, MA). Nitrogen content (%) was 0.0 0.00 SD for the white inorganic sand, 0.05 0.005 for the *Picea* wood, and 1.5 0.02 SD for nitrogen-rich soil OM. The nitrogen-rich soil OM was obtained from bags of commercial potting soil (“Nature’s Care, organic & natural potting mix”, Scotts Miracle-Gro, Marysville, OH, USA). After 14-months of colony growth, all vials were opened, termites were counted, and total termite colony biomass was measured. Colony survival rates were compared using a χ^2^ test. Failed incipient colonies (missing at least the king or the queen) were then excluded from the rest of the data analysis.

After all colonies were processed, a follow up experiment was performed to determine if subsequent colony growth was affected when access to nitrogen-rich soil OM was removed at the time when colonies enter their ergonomic growth phase^35,48^. Among the 30 successful incipient colonies reared on soil OM after 14 months, colonies were transferred to 1.5 L plastic containers (17.5 × 12.5 × 7 cm^3^, Pioneer Plastics, Dixon, KY) with either ~ 300 g white sand (n = 15) or ~ 100 g soil OM (n = 15) (same volume but different densities). All 14 month-old colonies of origin were ranked by colony size, the two largest were each randomly assigned to one of the two treatments, and the process was repeated with the two next ranked colonies until 15 colonies were assigned per treatment, resulting in treatment groups with equivalent colony size and variance. Each container was provisioned with three pieces of wood (*Picea sp.* 1.5 × 3.8 × 15 cm each) and moistened with water. Containers were then left for colony development with minimal disturbance, as colonies were checked twice a month for moisture content, and water was sprayed on the surface if need. After 6 months of subsequent colony development (= 20 month-old colonies) colonies were fully processed to count the total number of termites and measure the total biomass. For both experiments, all variables were compared using a student *t* test (α = 0.05), with soil treatment as a factor (sand *vs.* soil OM).

### Elemental analysis

All termites from three randomly selected 20 month-old colonies per soil treatment were dried at 60 °C for three days. The dry weights were recorded for each caste, and termites were ground using a micropestle. An aliquot of each sample was subjected to elemental analysis in a CHN analyser (Perkin Elmer, Waltham, MA, USA). Resulting nitrogen percentages were applied to the dry weights of each caste, and total nitrogen present in each colony was extrapolated. In addition, three 1 g samples each of the soil OM and the sand used in this study were subjected to elemental analysis. Differences in nitrogen present in termite colonies were subjected to a Student’s *t* test (α = 0.05) using soil treatment as a factor.

### Nitrogenase expression assay

Foraging populations from four field colonies of *C. formosanus* were collected in Broward Co. FL. The colonies were maintained in petri dishes (10 cm diameter × 1.5 cm high) (Fisher Scientific, Pittsburgh, PA, USA) with moistened Whatman #1 filter paper (Buckinghamshire, UK) for three days in order to vacate worker hindguts of field derived food, and homogenized gut content. Each colony was then separated into two groups of approximately 100 individuals and on a substrate of wood and either soil or sand as previously described. After four weeks, ten random workers from each colony and rearing condition were dissected and total hindgut RNA was extracted using the methods outlined in Peterson and Scharf^71^. Total cDNA was constructed using a ProtoScript II First Strand cDNA Synthesis Kit (New England Biolabs Inc Ipswitch, MA, USA).

The gene coding for the nitrogenase subunit (*nifH*) was selected as a target as it is highly conserved across diazotrophic organisms, and universal primers are well studied and available^72^. Quantitative PCR was conducted using cDNA as the template. Primers PolF and PolR^73^ were used to amplify the *nifH* gene. Three prokaryotic reference genes (*rpoA, rpoB, tuf*) were used for normalization of expression rates^74–76^, (Table 3). All qPCR assays were conducted in 20 µl reactions composed of 1 µl cDNA template, 50% SsoFast EvaGreen with Low ROX Supermix (Bio Rad, Hercules, CA, USA), 2% polyvinyl pyrrolidone (MW 40,000) (PVP-40), and 0.5 µM of each primer. Nuclease free water was added to bring the total reaction volume to 20 µL. The reactions and analysis were conducted on a QuantStudio 6 Flex System (Applied Biosystems, Forster City, CA, USA) Thermal cycling conditions for PCR reactions were as follows: initial denaturation for 2 min at 95 °C followed by 40 cycles of 15 s denaturation at 95 °C, 30 s. annealing at 56 °C (58 °C for the three reference genes), 30 s of extension at 72 °C. Resulting qPCR products were confirmed through gel electrophoresis indicating a distinct line at the target size. Ct values resulting from QPCR reactions were used to calculate the relative fold change (2^−Ct^ values) of nitrogenase expression in termites reared on soil versus those reared on sand^77^.

**Table 3:** Primers and PCR conditions used in this study.

Primer set	Target gene	Forward sequence	Reverse sequence	Annealing temp (°C)	Extension time (s)
Pol	*nifH*	TGCGAYCCSAARGCBGACTC	ATSGCCATCATYTCRCCGGA	56	30
rpoA	*rpoA*	GGTCGACGAACTGGAACTGT	CATCTCCTGCTCGGTCTTCT	58	30
rpoB1698f.	*rpoB*	AACATCGGTTTGATCAAC	CGTTGCATGTTGGTACCCAT	58	30
EF-Tu	Tuf	GTCATTCTGGTAGTTTCCGC	GAACCTCAAGCTCAACCAG	58	30
